# Suboptimal micronutrient intake among children aged 6 to 23 months in Ethiopia: Further analysis of the demographic and health survey

**DOI:** 10.1371/journal.pone.0305232

**Published:** 2024-07-09

**Authors:** Samrawit Mihret Fetene, Wubshet Debebe Negash, Tsegaw Amare, Tadele Biresaw Belachew, Banchlay Addis, Desale Bihonegn Asmamaw, Atitegeb Abera Kidie, Abel Endawkie, Sisay Maru Wubante, Alebachew Ferede Zegeye, Tadesse Tarik Tamir, Elsa Awoke Fentie

**Affiliations:** 1 Department of Health Systems and Policy, Institute of Public Health, College of Medicine and Health Sciences, University of Gondar, Gondar, Ethiopia; 2 Department of Reproductive Health, Institute of Public Health, College of Medicine and Health Sciences, University of Gondar, Gondar, Ethiopia; 3 School of Public Health, College of Health Science, Woldia University, Woldia, Ethiopia; 4 Department of Epidemiology and Biostatistics, School of Public Health, College of Medicine and Health Science, Wollo University, Woldia, Ethiopia; 5 Department of Health Informatics, Institute of Public Health, College of Medicine and Health Sciences, University of Gondar, Gondar, Ethiopia; 6 Department of Medical Nursing, School of Nursing, College of Medicine and Health Sciences, University of Gondar, Gondar Ethiopia; 7 Pediatric and Child Health Nursing, University of Gondar, Gondar, Ethiopia; Curtin University, AUSTRALIA

## Abstract

**Background:**

The recommended essential micronutrient such as food rich in vitamin-A or iron, multiple micronutrient powder or iron supplement, routine daily consumption of iodine, and vitamin-A supplement are deficient among children in Ethiopia. This has been a significant public health problem despite the government efforts. Although few studies have examined the micronutrient intake among children, they are limited in scope and methodological measurements. Analyzing the micronutrient intake among children across all regions and leveraging all essential micronutrient elements are crucial for generating improved evidence to better inform policy. Thus, we examined the micronutrient intake among children aged 6 to 23 months in Ethiopia.

**Methods:**

We used data from the Ethiopian Demographic and Health Survey. A two-stage stratified sampling technique was employed, and 1392 children aged 6 to 23 months were included in our analysis. We conducted a multilevel mixed-effect binary logistic regression analysis to identify determinants of micronutrient intake. In the final model, we used a p-value of less than 0.05 and Adjusted Odds Ratio (AOR) with their 95% confidence interval (CI).

**Results:**

We found that only 27.6% (95% CI: 26.8–31.6) of children aged 6 to 23 months were received the recommended micronutrients in Ethiopia. We identified that maternal educational status (Educated mothers (AOR = 2.09, 95%CI:1.23–3.58)), health facility delivery (AOR = 2.14, 95%CI:1.42–2.98), household wealth status (middle quantile (AOR = 1.80, 95%CI:1.01–3.21)), children’s age (12 to 23 months age (AOR = 2.36, 95% CI: 1.33–4.21)), and mother’s exposure to media (AOR = 1.70, 95%CI: 1.42–2.04) were increased micronutrient intake, whereas residing in the rural communities (AOR = 0.27, 95%CI: 0.21–0.34) decreased micronutrient intake.

**Conclusions:**

Nearly three-fourths of children aged 6 to 23 months did not receive the recommended essential micronutrients in Ethiopia. Therefore, there is a need to broaden strategies aimed at enhancing the intake by improving information and knowledge dissemination among mothers during facility visits and through media channels.

## Introduction

The first two years of life are a critical developmental stage that plays a crucial role in immune and physiological function [1]. However, during this period, children are most vulnerable to undernutrition due to factors such as low dietary intake, food inaccessibility, nutritional taboos, and infectious diseases [2]. Micronutrient deficiencies among children remain significant public health concerns in Ethiopia [3]. The most common micronutrient deficiencies include iron, iodine, vitamin A, and zinc [4]. All these conditions are responsible for more than half of all deaths in children under-five globally [5]. Furthermore, micronutrient deficiencies contribute to weakened resistance to infectious, intellectual disabilities, decreased nutrient uptake and delayed or impaired physical, mental and psychomotor development [6–8].

In Ethiopia, from 1990 to 2017, the prevalence of dietary iron, vitamin A, and iodine deficiency decreased by 20.1%, 16.7%, and 91.6%, respectively [9]. The National Nutrition Program (NNP II) is committed to implementing nutrition interventions to end hunger by 2030, including efforts to mitigate and prevent micronutrient deficiencies [10]. Despite governmental efforts, significant indicators of micronutrient intake remain low among young children. For instance, 29% of children aged 6 to 23 months consumed vitamin A-rich foods, while 24% consumed iron-rich foods [11]. In addition, only 6% of children aged 6 to 59 months received iron supplementation, and 53% consumed vitamin A supplementation [11]. Therefore, the country still suffers from micronutrient deficiencies [9].

The intake of micronutrients among children has been observed across different parts of the country [12–14]. These studies focused on each micronutrient intake separately, such as consumption of VA-rich food [14,15], intake of VA-supplements [16,17], and consumption of iron-rich foods [13]. The identified individual variables include education status, wealth status, occupation, age of the child, place of delivery and media exposure [12,14,18,19]. In addition, residence, region, and exposure to community-level media are among the community-level factors that have been identified [12,14,20]. Nonetheless, there is limited comprehensive evidence available regarding the status of micronutrient intake among children in Ethiopia, highlighting the need for further studies.

While various studies have attempted to examine the determinants of micronutrient intake separately, one study in Ethiopia assessed the micronutrient intake among children aged 6 to 23 months [12]. However, the study included only the emerging regions of Ethiopia (Afar, Benishangul Gumuz, Gambela, and Somali). These regions are characterized by scattered pastoralist and semi-pastoralist societies facing severe poverty [21], which makes it challenging to draw conclusions about the findings for the entire country. Furthermore, while previous studies have measured micronutrient intake, they have included deworming, but have not considered iodine utilization status. Therefore, previous studies are limited in scope and methodological measurements. Analyzing the micronutrient status among children across all regions and leveraging all essential micronutrient elements are crucial for generating improved evidence to better inform policy. Thus, we examined the micronutrient intake status among children aged 6 to 23 months in Ethiopia.

## Methods

### Study design, setting and period

This study used 2016 Ethiopian Demographic and Health Survey (EDHS) data collected through a community-based cross-sectional study design. The EDHS is a nationally representative household survey conducted every five years by the Central Statistical Agency of Ethiopia [22]. Ethiopia comprised nine regional states and two city administration (Addis Ababa and Dire Dawa) at the time of the survey. The nine regions can be divided into emerging regions (Afar, Benishangul-Gumuz, Gambela, and Somali) and developed regions (Tigray, Amhara, Oromia, Southern nationalities and peoples of Ethiopia, and Harari) [21,23–25]. The developed regions are mostly urbanized and have better infrastructure, healthcare access, education and transportation access than emerging regions [26]. Ethiopia remains one of the low-income countries in the region. Despite the importance of agriculture to the economy, the sector is marked by weak markets, limited access to advanced technologies, and a heavy reliance on erratic rainfall [27,28]

### Sampling procedure and sample size

A two-stage stratified sampling technique was applied to the EDHS 2016, using the 2007 Population and Housing Census as a sampling frame [22] to select the study participants. In the first stage of selection, the primary sampling units were chosen with a probability proportional to their size within each stratum. The primary sampling units in the survey are typically census enumeration areas (EAs), which are used to form clusters. Accordingly, a total of 645 EAs (202 in urban areas and 443 in rural areas) that represent the country were selected in the first stage.

In the second stage, a complete household listing was conducted in each of the selected clusters. Then, on average 28 households per EAs were selected by equal probability systematic sampling in the selected cluster. The overall selection probability for each household in the sample is the probability of selecting the cluster multiplied by the probability of selecting the household within the cluster. The details of the data collection and sampling procedure were presented in the full EDHS 2016 report [29]. Therefore, a total of 1392 weighted living children aged 6 to 23 months, living with their mothers, were included in this study from the selected EAs.

### Data source and populations

We used the kids recode (KR file) and household recode (HR file) data sets. We merged the two files using the cluster number to obtain the variable that measures routine daily iodine intake, which is in the HR file. The source population comprised all living children aged 6 to 23 months, whereas the study population consisted of all sampled children aged 6 to 23 months living with their mothers. Children living elsewhere were excluded from the analysis.

### Measurement of variables

The dependent variable was micronutrient intake among children aged 6 to 23 months. It is binary, coded as "1" ("Optimal") if the child had consumed at least three of the minimum recommended micronutrients. If the children received less than three of the minimum recommended micronutrients, it is coded as "0" ("Suboptimal"). The minimum recommended micronutrients included six options: food rich in VA or iron in the last 24 hours, multiple micronutrient Powder or iron supplement consumed within the previous seven days, routine daily consumption of iodine, and VAS within the last six months [10] (see Table 1).

**Table 1:** Variable measurement in the study of suboptimal micronutrient intake among children.

Level	Variables		Measurement
Outcome variable	Micronutrient intake	Foods rich in vitamin A	The consumption of seven food groups within a 24-hour period. These food groups included: i. Eggs, ii. Meat (beef, pork, lamb, chicken), iii. Pumpkin, carrots, and squash, iv. Any dark green leafy vegetables, v. Mangoes, papayas, and others fruits rich in VA fruits, vi. Liver, heart, and other organs and vii. Fish or shellfish. Accordingly, if the respondent reported that the child had consumed at least one of these, it was considered "yes"; otherwise, it was considered "no" for VA-rich food.
		Foods rich in iron	The consumption of four iron-rich food groups within a 24-hour period. These groups were i. Eggs, ii. Meat (beef, pork, lamb, chicken), iii. Liver, heart, and other organs, and iv. Fish or shellfish. Thus, if the respondent reported that the child had consumed at least one of these, it was considered "yes"; otherwise, it was considered "no" for iron-rich food.
		Multiple micronutrient powders	Multiple micronutrient powder was assessed by asking the respondents whether their child had received multiple micronutrient powder in the previous seven days.
		Routine iodine consumption	A child living in a household with tested iodized salt is utilizing iodine.
		Vitamin A consumption	VAS consumption for children aged 6 to 23 months in the last six months was assessed by reviewing the integrated child health card, and from the mother’s verbal response.
		Iron supplementation	Iron supplementation was assessed by asking the respondents whether their child had received iron pills or sprinkles with iron, or iron syrup in the previous seven days.
Individual level	Age of the mother	The age of mother was recoded as follows: “15–24 years” = “0”, “25–34 years” = “1” and “35 and above” = “2”.	
	Religion	The religion was recoded as follows: “Muslim” = “0”, “Orthodox” = “1”, “Protestant” = “2”, “Other” = “3”, including Catholic and traditional regions”	
	Educational status of mother and husband	The educational status of both the mother and husband was recoded as follows: "uneducated” = “0" and "educated” = “1”. The "educated" category included respondents who had attended primary school, secondary school, or higher education. On the other hand, individuals who had not received any formal education were recoded as "uneducated".	
	Current marital status	The current marital status variable was recoded as follows: "unmarried” = “0” and "married” = “1”. The "unmarried" category encompassed respondents who were single, widowed, divorced, or separated, while others were considered "married".	
	Childbirth size	Childbirth size was measured by assessing maternal perceptions of their baby’s size at birth for all live births that occurred during the last 5 years preceding the survey. Mothers were retrospectively asked to classify their babies’ sizes at birth as very large, larger than average, average, smaller than average, or very small. Subsequently, we recoded them into three categories: large (very large and larger than average), average, and small (smaller than average and very small).	
	Mother’s media exposure	Mother’s media exposure was assessed using three variables: listening to radio, watching television, and reading a newspaper, and labeled as “yes” if a woman had exposure to either of the three media sources at least once a week, or “no” if a woman had exposure to none of them.	
	Wealth index	The wealth index was utilized as a measure of each household’s socioeconomic standing. It assessed households based on the range and quantity of consumer goods they possessed, including items like televisions, bicycles, or cars, as well as housing features such as water sources, flooring materials, and sanitation facilities. Through principal components analysis, each household asset received a weighted score, reflecting its contribution to the household’s overall wealth status. These scores were then standardized to a standard normal distribution, resulting in a mean of zero and a standard deviation of one. Following standardization, each asset received a standardized score based on the household’s ownership status. These scores were aggregated for each household, yielding a total score used to rank individuals within the sample. Subsequently, the population sample was divided into quintiles, each comprising 20% of the population. The bottom 20% quintile represented the poorest households, followed by the next 20% for those considered poor, another 20% for the middle-class, and the top 40% for wealthy and wealthiest households [30].	
Community level	Community level media exposure and community level poverty	Community-level media exposure and community-level poverty variables were not directly found in the survey; however, these variables might be significant determinants of micronutrient intake among children. Therefore, we created them by combining individual-level variables, using average approaches to conceptualize the neighborhood effect on micronutrient intake. A community-level media exposure variable is measured by the proportion of mother who have been exposed to at least one media and categorized based on the median value as low (communities with <50% of mother exposed) and high (communities with ≥50% of mother exposed) since proportion of media exposure level among mother was not normally distributed. On the other hand, community level poverty is the level of poverty in the community was determined by the proportion of households in the poorer and poorest quintiles, as derived from the wealth index results. Similarly, we categorized the community level poverty exposure proportion into low (if the proportion of households in the poorer and poorest quintiles <50%) and high (if the proportion >50%) using the median.	

The independent variables for this study were categorized into individual and community variables (see Table 1). The individual level variables includes the age of the mother, mother’s and husband’s educational and occupational status, sex of household head, religion, marital status, wealth index, age of the child, occurrence of diarrhea, occurrence of a cough, number of antenatal care visit (ANC), postnatal care (PNC) checkup, place of delivery, current pregnancy status and childbirth size [12–14,17–19]. On the other hand, place of residence, community-level media exposure and community-level poverty were the community-level variables [30–32].

### Data management and analysis

The data were extracted, cleaned, recoded, and analyzed using STATA version 16. Sampling weights were applied during statistical analysis to address the unequal probability of selection inherent in the sampling design used in DHS data. Descriptive statistics were presented using tables, graphs and narrations.

Given that the DHS data has a hierarchical structure which violates the independence assumption of the standard logistic regression model, we utilized a multilevel logistic regression analysis. Moreover, children from the same cluster are more likely to be similar than those from different clusters. This highlights the importance of accounting for between-cluster variability by employing advanced models. The Interclass Correlation Coefficient (ICC) was then checked to assess whether significant clustering was present [33]. An ICC greater than 10% is indicates eligibility for multilevel analysis [34]; in our study, the ICC was found to be 79%.

In this multilevel analysis, we fitted models; the Model I- a model without explanatory variables; Model II- a model with individual-level variables and Model III- a model with both individual and community-level variables, simultaneously. Since the models were nested, model comparison and fitness were assessed based on the Likelihood Ratio test [35], and deviance (-2*LLR) values. The model with the lowest deviance (Model III) was selected as the best-fitted model. In the bivariable analysis, variables with a p-value < 0.2 were considered for multivariable analysis. Finally, adjusted odds ratios with 95% CI and a p-value of ≤ 0.05 in the multivariable analysis were used to declare statistically significant determinants of micronutrient intake. The variance inflation factor (VIF) was used to test multicollinearity. There was a VIF of less than five for each independent variable with a mean VIF of 1.51, indicating no significant multicollinearity between independent variables.

### Ethical approval and consent to participate

The study utilized a secondary analysis of publicly available DHS data, which does not contain personal identifier. Permission to access the data was obtained from the MEASURE DHS program (available from https://www.dhsprogram.com/Data/). To conduct our study, we completed the registration process and formally requested the dataset from the DHS online archive. Subsequently, on February 6, 2023, we obtained approval to access and download the data files. The data collection adhered to international and national ethical guidelines and received ethical approval for the survey from the Ethiopian Public Health Institute, the National Research Ethics Review Committee, and the Institutional Review Board for ICF Macro International.

## Results

### Socio-demographic and economic characteristics of respondents

Table 2 describes the socio-demographic and economic characteristics of the study participants. Nearly 54% of mothers of children were in the age group of 35 years or older. The mean age of the mother was 36 years (SD ± 11.48). Two-thirds of the respondents had no formal education, and 39% of them were in the very poor wealth quintiles.

**Table 2:** Socio-demographic and economic characteristics of respondents in Ethiopia, EDHS 2016 (n = 1392).

Variables	Weighted frequency	Percentage (%)
Age (in years)		
15–24	116	8.33
25–34	524	37.64
> = 35	752	54.03
Religion		
Muslim	780	56.03
Orthodox	328	23.56
Protestant	252	18.10
Other^a^	32	2.40
Mother’s educational status		
Uneducated	919	66.02
Educated	473	33.98
Husband’s educational status		
Uneducated	711	53.10
Educated	628	46.90
Mother’s occupational status		
Unemployed	888	63.79
Employed	504	36.21
Husband’s occupational status		
Unemployed	215	16.06
Employed	1124	83.94
Current marital status		
Not married	53	3.81
Married	1339	96.19
Wealth index		
Very poor	547	39.32
Poor	273	19.62
Middle	193	13.86
Rich	284	20.40
Very rich	95	6.82
Sex of household head		
Male	1093	78.52
Female	299	21.48
Media exposure		
No	983	70.6
Yes	409	29.4

*Other* ^*a*^ *= Catholic*, *traditional*.

### Child and obstetric related characteristics

Among the children included in the study, 57.26% were aged 13 to 23 months, and approximately 40% had an average weight at birth. The findings showed that 16.88% and 18.18% of children had reported diarrhea and cough, respectively, in the last two weeks preceding the survey. In addition, 62.96% of mothers had received ANC; 31.29% delivered at health facilities, and 6.69% had PNC checks within two months after delivery (see Table 3).

**Table 3:** Child and obstetric related characteristics, EDHS 2016 (n = 1392).

Variables	Weighted frequency	Percentage (%)
Age of child		
6–12 months	595	42.74
13–23 months	797	57.26
Childbirth size		
Large	416	29.89
Average	552	39.66
Small	424	30.45
Had diarrhea recently		
No	1157	83.12
Yes	235	16.88
Had cough in last two weeks		
No	1139	81.82
Yes	253	18.18
Number of ANC visit		
Less than 4 visits	493	37.04
Four or more visits	838	62.96
PNC checkup		
No	1242	93.31
Yes	89	6.69
Place of delivery		
Home	955	68.61
Health facility	437	31.39
Current pregnancy status		
Non-pregnant	1291	92.74
Pregnant	101	7.26

### Community level variables

More than three fourths (86.06%) of the respondents were rural dwellers, and 65.30% were in higher community poverty (see Table 4).

**Table 4:** Community-level variables in Ethiopia, EDHS 2016 (n = 1392).

Variables	Frequency	Percentage (%)
Residence		
Urban	194	13.94
Rural	1198	86.06
Community-level media exposure		
Low	904	64.94
High	488	35.06
Community-level poverty		
Low	483	34.70
High	909	65.30

### Micronutrient intake among children

**Fig 1:**
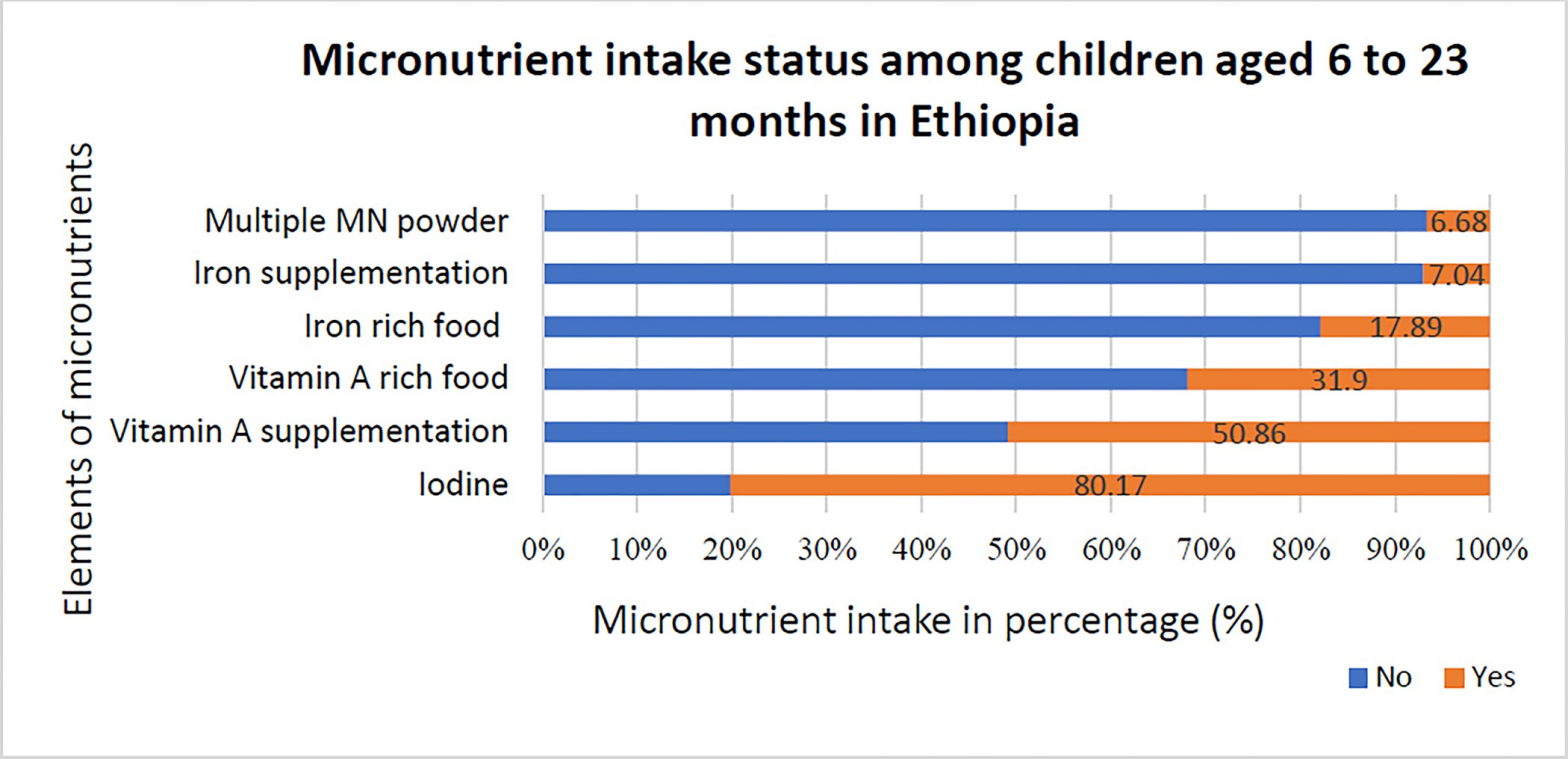
Micronutrient intake s from each element of micronutrient among children aged 6 to 23 months, EDHS 2016.

The overall micronutrient intake among children aged 6 to 23 months in Ethiopia was 27.6% (95%CI: 26.8, 31.6). Nearly 72.4% of children aged 6 to 23 months had suboptimal micronutrient intake. More than half (50.86%) of the children had received VAS within the previous six months, and 80.17% lived in households with tested iodized salt. Moreover, nearly 32% of children had consumed foods rich in VA and 17.89% had consumed foods rich in iron within the previous 24 hours. Regarding the multiple micronutrient powder, 6% of children had received it, and 7.04% had received iron supplements within the last seven days (see Fig 1).

### Measure of variation using random effects and model fitness

There was a significant variation in micronutrient intake among children aged 6 to 23 months across both individual and community levels. The ICC of micronutrient intake among children aged 6 to 23 months in the null model was 0.79, meaning 79% of the variation in micronutrient intake among children aged 6 to 23 months was due to the differences between clusters. Deviance was used to assess the model fitness, and the model with low deviance (Model III) was selected for the final analysis to identify the determinants of micronutrient intake among children aged 6 to 23 months in Ethiopia (see Table 5).

**Table 5:** Random-intercept model of multilevel analysis for micronutrient intake among children.

Measure of variations	Model I	Model II	Model III
LLR	-565.93	-472.42	-469.68
ICC	79.0	84.3	78.4
Deviance (-2*LLR)	1131.86	944.84	939.36

LLR = Log Likelihood Ratio.

ICC = Intraclass Correlation Coefficient.

Model I (Null model) = a model without independent variables.

Model II = a model with individual-level variables.

Model III = a model adjusted for both the individual and community-level variables.

### Determinants of micronutrient intake among children

Since model III was selected as the better-fitted model, variables such as mother’s educational status, wealth index, age of the child, place of delivery, media exposure, and residence were significant in this model (see Table 6).

**Table 6:** Multilevel mixed effect logistic regression analysis of determinants of micronutrient intake among children (n = 1392).

Variables	Micronutrient intake		COR (95%CI)	Model II AOR (95% CI)	Model III AOR (95% CI)
	Yes n (%)	No n (%)			
Age of mother					
15–24	34(29.3)	82(70.7)	0.98(0.64–1.50)	0.24(0.07–0.84)	0.28(0.09–0.89)
25–34	149(28.4)	375(71.6)	0.93(0.73–1.19)	0.51(0.28–0.93)	0.49(0.26–0.93)
> = 35	224(29.8)	528(70.2)	1	1	1
Mother educational status					
Educated	197(41.7)	276(58.4)	2.40(1.89–3.06)	2.26(1.33–3.83)	2.09(1.23–3.58) *
Uneducated	210(22.9)	709(77.4)	1	1	1
Husband educational status					
Educated	227(36.2)	401(63.9)	1.90(1.51–2.42)	1.07(0.41–2.80)	0.85(0.35–2.06)
Uneducated	163(22.9)	548(77.1)	1	1	1
Household wealth index					
Very rich	43(45.3)	52(54.7)	2.07(1.30–3.25)	0.83(0.31–2.23)	1.01(0.51–2.00)
Rich	112(39.4)	172(60.6)	4.63(3.21–6.64)	1.25(0.82–1.91)	0.86(0.73–1.01)
Middle	73(37.8)	120(62.2)	1.53(1.08–2.16)	2.18(1.13–4.22)	1.80(1.01–3.21) *
Poor	97(35.5)	176(64.5)	1.38(1.01–1.88)	1.10(0.71–1.73)	0.91(0.52–1.61)
Very poor	156(28.5)	391(71.5)	1	1	1
Place of delivery					
Health facility	181(41.4)	256(58.6)	2.28(1.79–2.91)	2.26(1.69–2.86)	2.14(1.42–2.98) *
Home	226(23.7)	729(76.3)	1	1	1
Current pregnancy status					
No pregnant	373(28.9)	918(71.1)	0.80(0.52–1.24)	0.50(0.49–0.51)	0.11(0.03–0.42)
Pregnant	34(33.7)	67(66.3)	1	1	1
Age of the child					
13 to 23 months	261(32.8)	536(67.3)	1.49(1.18–1.89)	2.41(1.35–4.32)	2.36(1.33–4.21) *
6 to 12 months	146(24.5)	449(75.5)	1	1	1
Childbirth size					
Large	150(36.1)	266(63.9)	2.06(1.54–2.84)	1.92(0.92–4.01)	1.38(0.63–3.04)
Average	166(30.1)	386(69.9)	1.57(1.18–2.10)	0.61(0.35–1.07)	0.35(0.12–1.06)
Small	91(21.5)	333(78.5)	1	1	1
Media exposure					
Yes	162(39.6)	247(60.4)	1.98(1.54–2.52)	1.91(1.63–2.26)	1.70(1.42–2.04) *
No	245(24.9)	738(75.1)	1	1	1
Residence					
Rural	305(25.5)	893(74.5)	0.31(0.23–0.42)		0.27(0.21–0.34) *
Urban	102(52.6)	92(47.4)	1		1
Region					
City administration	74(39.6)	113(60.4)	1.79(1.26–2.52)		0.10(0.05–2.22)
Developed regions	178(28.4)	449(71.6)	1.08(0.84–1.39)		0.71(0.42,1.21)
Emerging regions	155(26.8)	423(73.2)	1		1
Community-level media exposure					
High	175(35.9)	313(64.1)	1.62(1.28–2.05)		0.50(0.14–1.81)
Low	232(25.7)	672(74.3)	1		1

*Statistically significant at p-value <0.05 in the full model (model III).

AOR: Adjusted Odds Ratio, COR: Crude Odds Ratio, Model II: adjusted for individual-level factors, Model III: adjusted for both individual and community-level factors (full model).

Those children born from educated mothers were 2.09 (AOR = 2.09 95%CI: 1.23–3.58) times more likely to have micronutrient intake than children born from uneducated mothers. The odds of micronutrient intake among children who were delivered in a health facility were 2.14 (AOR = 2.14 95%CI: 1.42–2.98) times higher than those delivered at home. Children from households with the middle household wealth status were 1.80 (AOR = 1.80 95%CI: 1.01–3.21) times more likely to have micronutrient intake as compared to their counterparts. Those children who reside in rural communities were 73% less likely to have micronutrient intake than urban residents (AOR = 0.27 95%CI: 0.21–0.34). The odds of micronutrient intake among children aged 13 to 23 months were 2.36 (AOR = 2.36 95%CI: 1.33–4.21) times higher than children aged 6 to 12 months. Those children whose mothers had been exposed to media were 1.70 (AOR = 1.70 95%CI: 1.42–2.04) times more likely to have micronutrient intake than children whose mothers had no media exposure.

## Discussion

According to the national guidelines for prevention and control of micronutrient deficiency, all children need to receive micronutrients [10]. However, our study revealed that the overall micronutrient intake among children aged 6 to 23 months in Ethiopia was 27.6% (95%CI: 26.8–31.6). This implies that substantial proportion of children aged 6 to 23 months in Ethiopia are not meeting the recommended intake of micronutrients as outlined in the national guidelines, which places the country far from reaching its targets set under the NNP II.

Our finding was lower compared to another studies conducted in Ethiopia (37.3%) [12], Malawi (79.1%) [36], Rwanda (68%), Burundi (78%) [37], and India (58.1%) [38]. The observed differences in these studies might be due to variations in the measurement of the outcome variable. For instance, in the study done in Ethiopia [12], children were considered to have received micronutrient if they had consumed at least one of the minimum recommended micronutrients. In contrast, our study defines a child as having received a micronutrient if they have consumed a minimum of three out of the recommended micronutrients. Other reasons for observed differences could include variations in the availability of dense forests and water reservoirs [30], cultural belief that children may have difficulty chewing and digest animal products [39], and increases in food prices [40]. Furthermore, there are differences in study areas and participants; a study done in Ethiopia [12] considers only study participants who reside in emerging regions, whereas our study includes children from all regions. There was a participant age category difference also; most studies consider children aged less than five years [38,41,42], while we consider children aged 6 to 23 months.

This study showed that children whose mothers were educated had higher levels of micronutrient intake than their counterparts. This finding is consistent with studies done in Ethiopia [19], Indonesia [43], Uganda [44], Pakistan [45], and Sri Lanka [46]. This might be because educated mothers are easier to familiarize themselves with new information and knowledge, and they know the importance of the appropriate child-feeding practices [19]. This finding implies that mother’s educational status has a positive association with their children’s micronutrient intake. As result, the government should establish interventions aimed at improving those practices, that target families with low levels of education and design promotional materials that take low parental levels of education into account.

The odds of micronutrient intake for children aged 13 to 23 months was higher than those aged 6 to 12 months. Similarly, studies conducted in Ethiopia [12], Ghana [47], and Pakistan [48] revealed that children aged 13 to 23 months were more likely to receive micronutrients. This could be because mothers believe that younger children’s intestines are incapable of digesting certain foods, such as eggs, green vegetables, and meat, and they do not give them to their children [49]. This implies that children between the ages of 6 to 12 months require specific attention to enhance their micronutrient intake. Therefore, it would be better for the government to establish a special program focused on improving mothers’ perceptions and behaviors to reduce negative traditional beliefs that might contribute to low micronutrient intake among children aged 6 to 12 months.

Children from the middle household wealth status were more likely to have micronutrient intake than their counterparts. This study is supported by other studies done in Ethiopia [13,50], Ghana [51], and Philippines [52]. This could be due to the households’ low wealth status, which has been linked to food insecurity and, as a result, a lack of micronutrient-rich food consumption [53]. In addition, the high cost of foods is seen as an important barrier to the consumption of a nutritionally sound diet, and therefore poor households cannot afford the cost of these foods [54]. This finding implies that children from poor households may not meet the recommended consumption of micronutrients. Therefore, it is imperative for the government to implement initiatives aimed at empowering mothers to generate income, thereby overcoming food insecurity, and ensuring access to micronutrient-rich foods for their children. In contrast, a study done in Tanzania showed no differences in micronutrient intake based on household wealth status [55].

Children whose mothers had been exposed to media were more likely to have micronutrient intake than their counterparts. This finding is in line with studies done in Ethiopia [30–32], Nigeria [56], Indonesia [57] and Nepal [58]. This could have occurred due to national radio and television promotions of child nutrition-related media advertisements [30]. This indicates that creating media advertisements emphasizing the importance of micronutrient intake for children, along with promoting the recommended amount, can significantly contribute to improving children’s intake of micronutrients. Hence, the government should consider expanding the existing program, which aims to promote child nutrition-related practices using mass media, to enhance mothers’ awareness of the importance of micronutrients.

The odds of micronutrient intake among children delivered in a health facility were higher than those delivered at home. This finding is supported by another study done in Ethiopia [42]. One possible explanation is that mothers who give birth in a hospital receive more information and knowledge about their children’s nutrition practices from their healthcare providers during follow-up. Considering this, the Ethiopian government should contemplate its multidimensional strategies to improve health facility delivery through health extension workers. Besides improving maternal health, this will also contribute to enhancing the micronutrient intake status of the children.

Those children who reside in rural communities were less likely to have micronutrient intake than urban residents. This finding is in line with a study done in Ethiopia [12,59] and Nepal [60]. A possible explanation could be that rural mothers lack awareness of infant and young child feeding practices due to insufficient counseling on feeding practices and limited access to health services in rural areas [59]. In contrast, studies done in Nigeria [61], Ghana [62] and India [63] revealed that children born to mothers that lived in rural areas were more likely to get dietary diversity than those in urban residences. The potential explanation might be that food fortification and supplementation initiatives are more targeted towards rural areas [12]. Moreover, mothers in rural areas often have backyard gardens, which improve access to local foods and influence their children’s eating habits [64].

### Strengths and limitations of the study

This study addressed the identified limitations in scope and methodology of a similar study conducted in Ethiopia. Our study was conducted using weighted nationally representative data with a substantial sample size and multilevel analysis was employed to account for the hierarchical nature of the DHS data, ensuring reliable standard error and estimates. Given its reliance on national survey data, this study can provide valuable insights for policymakers and program planners to design appropriate interventions at both national and regional levels.

However, the study findings are interpreted with consideration of limitations. Since data were collected through respondents’ self-reporting, there may be a possibility of recall bias. We did not include supply-side determinants of outcome variables due to the use of secondary data, and there was no attempt to adjust for them because data on these variables were not collected. The study also might be prone to social desirability bias, as individuals might respond to questions in a manner deemed socially acceptable rather than expressing their true feelings. While there is a possibility of overestimating the results, it is important to note that the data collectors underwent comprehensive training, and the DHS questionnaires were appropriately designed, which may help mitigate the impact of social desirability bias. Furthermore, we utilized an outdated method for variable reduction, which we consider to be a limitation of the study.

## Conclusion and recommendation

Nearly three-fourths of children aged 6 to 23 months did not receive the recommended essential micronutrients in Ethiopia. Despite the government’s efforts to implement the National Nutrition Program (NNP I and NNPII), our study findings indicate that significant efforts are still required to address the suboptimal micronutrient intake among children aged 6 to 23 months in Ethiopia. Therefore, there is a need to broaden strategies aimed at enhancing the intake by improving information and knowledge dissemination among mothers during facility visits and using media channels.
